# Diet Supplementation Influences Ghrelin System Expression in the Skin Appendages of the Sheep

**DOI:** 10.3390/vetsci12010041

**Published:** 2025-01-10

**Authors:** Margherita Maranesi, Cecilia Dall’Aglio, Sara Moscatelli, Elisa Palmioli, Paola Coliolo, Daniele Marini, Gabriella Guelfi, Paola Scocco, Francesca Mercati

**Affiliations:** 1 Department of Veterinary Medicine, University of Perugia, Via San Costanzo 4, 06126 Perugia, Italy; margherita.maranesi@unipg.it (M.M.); elisa.palmioli@dottorandi.unipg.it (E.P.); paola.coliolo@unipg.it (P.C.); daniele.marini@dottorandi.unipg.it (D.M.); gabriella.guelfi@unipg.it (G.G.); francesca.mercati@unipg.it (F.M.); 2 School of Biosciences and Veterinary Medicine, University of Camerino, Via Pontoni 5, 62032 Camerino, Italy; sara.moscatelli@unicam.it (S.M.); paola.scocco@unicam.it (P.S.); 3 International School of Advanced Studies, University of Camerino, Via Madonna delle Carceri 9, 62032 Camerino, Italy; 4 Department of Philosophy, Social Sciences, and Education, University of Perugia, Piazza G. Ermini, 1, 06123 Perugia, Italy; 5 Department of Organismal Biology, Evolutionary Biology Centre, Uppsala University, Norbyvägen 18A, 75236 Uppsala, Sweden

**Keywords:** ghrelin receptor, hair follicle, integumentary system, immunohistochemistry, real-time PCR

## Abstract

Ghrelin (GhRL) is an orexigenic hormone influenced by nutritional state. It plays a role in skin repair and diseases, though little information exists regarding its function in this organ. GhRL and its receptor were investigated in the skin of sheep under different feeding conditions to explore GhRL system presence and possible modifications due to diet. Three-year-old female sheep were free to graze from June to the pasture maximum flowering (MxF group) and from this period to maximum dryness addicted (Exp group) or not (MxD group) with 600 gr/die/head of barley and corn. Skin samples were processed for immunohistochemistry and real-time PCR. The immunostaining showed the presence of the GhRL system in skin appendages. Indeed, the ligand was localized in the hair follicles whereas the receptor was also observed in sweat glands and smooth muscle cells. The expression of both genes was significantly higher in the Exp group (3.6 and 2.9 folds respectively, *p* < 0.05) compared with the MxF group. These results suggest that the GhRL system is involved in the regulation of hair follicles and sweat glands. In addition, diet supplementation may positively modulate the expression of GhRL and its receptor in the skin.

## Simple Summary

Semi-natural pastures provide the primary food source for sheep, whose grazing activity contributes to preserving grassland biodiversity. However, summer drought stress reduces the nutritional value of pastures, with negative consequences on the morpho-functional characteristics of the digestive system and other sheep systems. Understanding the molecules linked to energy metabolism is important for identifying suitable markers of tissue functionality and ensuring animal well-being, a key factor in sustainable livestock production. Ghrelin plays a role in food intake and is involved in tissue regeneration, repair and diseases at the skin level, even if information about this organ is very scarce. This study aims to analyze the ghrelin system in the skin of sheep grazing on seminatural pasture during the spring–summer season and compare differently fed animals. The results indicate that ghrelin and its receptor are localized in sheep skin appendices, with greater expression in animals fed with diet supplementation. This finding suggests that diet may influence the proliferative activity of the hair follicle through variations in the ghrelin system expression. These insights could be a useful tool for the breeders to counteract the negative effects of increasing summer aridity.

## 1. Introduction

Ghrelin (GhRL) is an acylated peptide encoded by the preproghrelin gene [1]. It consists of 28 amino acids in humans and rats [1] but only 27 amino acid peptides in bovines and sheep due to the absence of the glutamine residue 14 [2]. GhRL exists in two forms: the non-acylated and the acylated GhRL; the latter represents the active form with a fatty acid side chain at its third serine residue [2]. GhRL was first purified from the rat stomach. The enteroendocrine cells in the mucosa of the fundus and body of the stomach identified as X/A-like cells are the main site of production of this hormone, in both rats and humans [3,4]. Other sites producing smaller amounts of GhRL include several hypothalamic nuclei [5], the pituitary gland [6] and various peripheral tissues [7] such as kidney, lung, placenta, testis, ovary, pancreas, adrenal gland, muscle tissue and bone tissue. Thus, this molecule has a wide tissue distribution and participates in numerous central and peripheral actions.

Ghrelin stimulates growth hormone (GH) release, an effect that appears highly pronounced [7]. Its most recognized role is the regulation of food intake with an orexigenic effect, which induces an increase in appetite [8]. The stomach secretes GhRL in response to eating behavior. GhRL levels rise before meals, when the stomach is empty, and decrease about an hour after eating, as shown in humans and sheep [9,10]. Besides meal-related fluctuations, GhRL levels decrease in obesity and increase in lean individuals [11], suggesting a role in long-term energy homeostasis.

Beyond energy balance, GhRL is involved in the regulation of other physiological processes with central and peripheral effects. These include sleep modification [12], stress and anxiety management [13], suppression of brown fat thermogenesis [14] and cardiovascular functions such as vasodilatation and cardiac contractility [15]. GhRL also influences reproductive function through the hypothalamic-pituitary-gonadal axis. Furthermore, GhRL acts as a potent anti-inflammatory and antimicrobial mediator [16]. Hence, GhRL is a multifaceted peptide hormone [2] with diverse numerous functions depending on the tissue expressing the receptor.

Ghrelin binds to a specific G-protein coupled receptor identified in 1996 [17]. This receptor was initially described as the target for different synthetic GH secretagogues and was accordingly called the growth hormone secretagogue receptor (GHS-R) [17]. Two different splice forms of the receptor exist: GHS-R1a and GHS-R1b [17]. Type 1a is a full-length receptor containing 366 amino acids with seven transmembrane domains. Type 1b is a splice variant containing the second exon that lacks biological activity in GH-releasing or calcium-related functional assays [7]. The GHS-R is widely distributed in the central and peripheral tissues where it mediates the main GhRL-stimulated actions [18].

Ghrelin is involved in skin repair and the molecule was suggested as a potential endogenous anti-inflammatory and anti-fibrotic factor [19,20]. The skin is a metabolically active organ characterized by its own secretion and the pilosebaceous unit is the main site for hormone production [21]. The hair follicle is an appendage of mammalian skin which appears as an invagination of the epidermis into the dermis. It is associated with the sebaceous gland, the sweat gland and the arrector pili muscle [22]. The hair follicle has a peculiar cyclical activity that allows this organ to ensure the continuous renewal of the hair, a structure that protects the surface of the animal’s body. The gland secretions are poured into the hair canal and on the skin surface to improve skin barrier function. The study of the pilosebaceous unit and the molecules involved in its biology is of great interest in livestock animals, whose coat represents an important valuable product to be taken into consideration for the support and development of small farms

Since no information is available regarding the presence of GhRL and its receptor in the integumentary system of sheep, this study aims to investigate the presence of the GhRL system and explore whether it may change during the pasture vegetative cycle and may be influenced by alimentary supplementation.

## 2. Materials and Methods

**Figure 1:**
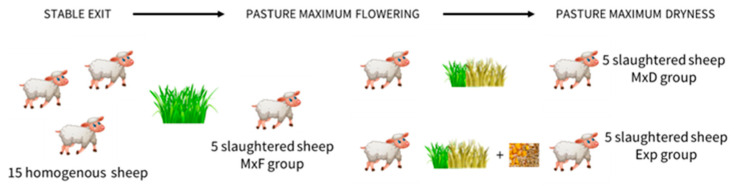
Experimental plan of feeding.

The experiment was performed on 15 Comisana x Appenninica adult female sheep reared on a secondary semi-mesophylous pasture in the Central Apennine belonging to the sub-Mediterranean climate [23]. The sheep were free to graze from June to the pasture maximum flowering established based on the anthesys of Graminaceae; the sheep were in the dry period and were homogenous in age (3 years), reproductive performance (2 pregnancies with 1 lamb each) and body condition score (1.9). At the end of this period (early July), 5 sheep were slaughtered (MxF group). The remaining sheep were divided into two groups and, until to the maximum dryness of pasture (early September), were fed ad libitum on pasture (MxD group) or with 600 gr/die/head of barley and corn (1:1) in addition to the pasture grass (Exp group), after which they were slaughtered (Figure 1) [24].

Experimental procedures were approved by the Ministry of Health (No. of approval 95/2018-PR). All animals were intended for human consumption and were slaughtered at the abattoir in accordance with the Council Regulation (EC) No. 1099/2009 on the protection of animals at the time of killing under law n.333/98 (Council Directive 93/119/EC of 22 December 1993) as specified by Annex C of Section II.

Skin samples were collected from the thoracic region and processed for immunohistochemistry and real-time PCR. Samples for histologic procedures were quickly fixed in a 10% neutral-buffered formalin solution in phosphate buffered saline (PBS 0.1M, pH 7.4) and then prepared for histological analysis. Samples for molecular biology were immediately frozen in liquid nitrogen and stored at −80 °C.

### 2.1. Immunohistochemistry

Immunohistochemistry was performed as previously described [25]. The skin samples from each sheep were embedded in paraffin wax and cut to obtain 5 µm thick sections. The skin sections were placed on poly-L-lysine-coated slides, deparaffinized, rehydrated through graded ethanol to distilled water and then dipped for 10 min in 3% H_2_O_2_ to reduce endogenous peroxidase activity. To perform antigen retrieval, sections were microwaved for 15 min at 750 W in citrate buffer solution (10 mM, pH 6). All the subsequent steps were carried out in a moist chamber at room temperature to prevent the evaporation of the reagents. Non-specific binding was blocked by 1:10 normal goat serum (Vector Laboratories, Burlingame, CA, USA), then the sections were incubated overnight at room temperature with the following polyclonal primary antisera: 1:400 rabbit anti-GhRL antibody (ab-129383, Abcam, Cambridge, UK) and 1:600 rabbit anti-GHS-R antibody (ab-188986, Abcam, Cambridge, UK). After rinsed with PBS buffer, the sections were incubated with a biotin-conjugated goat anti-rabbit IgG secondary antibody diluted 1:200 in PBS for 30 min (Vector Laboratories). The visualization of the reaction was performed by using an avidin-biotin system for 30 min (Vectastain ABC kit; Vector Laboratories) and 3,3′diaminobenzidine-4-HCL as chromogen (DAB, SK-4100, Vector Laboratories). The slides were counterstained with Mayer’s hematoxylin and mounted with Eukitt medium (Sigma-Aldrich, Alcobendas, Spain) for light microscopy. The sections were washed with PBS between all incubation steps, except after normal serum. Control sections of nonspecific staining were prepared by omitting the primary antibodies and incubating the slides with PBS or rabbit immunoglobulin G (I-1000-5, Vector Laboratories). All sections were observed under a photomicroscope (Nikon Eclipse E800, Nikon Corp., Tokyo, Japan) connected to a digital camera (Nikon Dxm 1200 digital camera) and analyzed by three independent investigators. Five randomly chosen microscopic fields for each section were evaluated and the mean staining intensity value was expressed in arbitrary units as follows: absent (0), weak (0.5), moderate (1), strong (2) and very strong (3) [26,27].

### 2.2. RNA Extraction and Real-Time PCR

The total RNA was extracted from the skin specimens of five ewes for each sheep group as previously described [28]. Genomic DNA contamination was prevented by treatment with deoxyribonuclease [29]. Five µg of total RNA were reverse transcribed in 20 µL of iSCRIPT cDNA using random hexamers, following the protocol provided by the manufacturer (Bio-Rad Laboratories, Milan, Italy).

The multiplex RT-PCR amplification was performed using 1.0 μL of cDNA as a template for GhRL, GHS-R, and β-Actin (ACTB) housekeeping primer (Table 1) [24]. Cycling conditions consisted of a denaturing cycle at 94 °C for 1 min and 15 s, followed by 30 cycles at 94 °C for 15 s, 62 °C for 30 s and 72 °C for 45 s and a final extension cycle at 72 °C for 10 min. For each PCR, a negative control without cDNA was included. The complete set of cDNA samples (five skin samples for each sheep group) was processed in a single PCR and each sample run in triplicate. The analysis of the amplified product was carried out as reported elsewhere [30,31]. Each sample was normalized to the geometric mean of one reference gene, ACTB [24].

### 2.3. Statistical Analysis

To assess the effects of pasture vegetative cycle and alimentary supplementation on ghrelin system expression, we employed a combination of parametric and non-parametric tests suited to the distribution characteristics of our data.

In particular, for the statistical evaluation of the graded scores given to different levels of immunohistochemical staining of ghrelin in hair follicles, a non-parametric Kruskal–Wallis test was conducted since the assumptions of normality for a parametric test were not met. Post hoc comparisons were then carried out using Dunn’s test with Holm correction.

For the ghrelin receptor, two separate one-way ANOVA tests were performed depending on the localization of the scored intensity: one for the ghrelin receptor in the hair follicle and another for the ghrelin receptor in the sweat glands. The ANOVA assumptions were checked using base R plot function. In both cases, the assumptions were satisfied. Tukey’s Honest Significant Difference (HSD) test was used for post hoc pairwise comparisons.

All analyses were performed in R (version 4.1.2, R Development Core Team 2023) using the base stats package.

The Shapiro–Wilk test was used to check the normality of data of GhRL and GHS-R gene expression, which were analyzed by the non-parametric Kruskal–Wallis test followed by the Student–Newman–Keuls test.

## 3. Results

### 3.1. Immunohistochemistry

The immunohistochemistry revealed a clear and intense immunostaining of the GhRL system in the skin appendages of the sheep, with the receptor showing distribution than the ligand.

**Figure 2:**
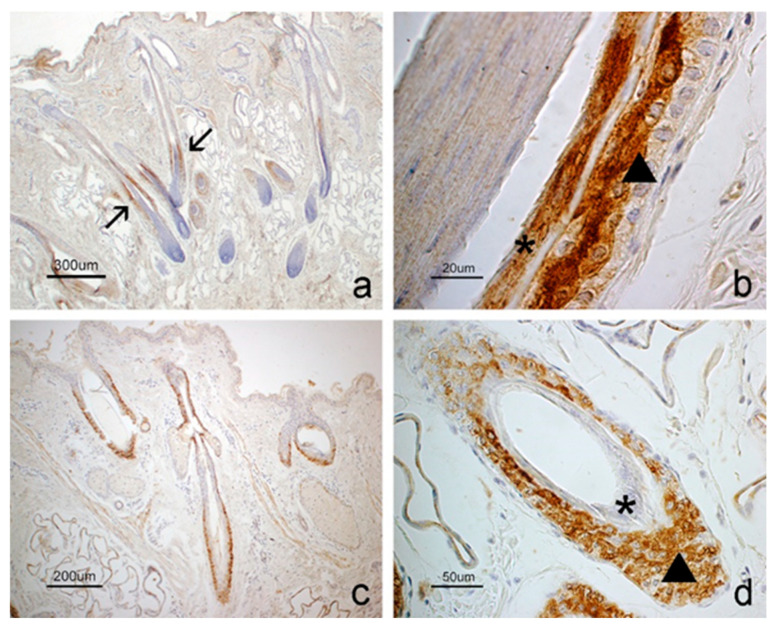
Immunostaining for (**a**,**b**) GhRL and (**c**,**d**) GHS-R in the hair follicles of the sheep. (**a**) Several hair follicles show the positivity to GhRL in the suprabulbar region (arrows) while the other parts of the structure are negative such as the infundibulum, isthmus and bulb. (**b**) GhRL is localized in the inner root sheath (asterisk) and in the suprabasal cells of the outer root sheath (arrowhead) while basal layer appears negative. (**c**) GHS-R immunostaining is distributed along the wall of the hair follicle in the foreground and in the infundibulum region of the two follicles placed on the sides while the epidermis is negative. (**d**) GHS-R immunostaining is localized in all layers of the ORS (arrowhead) but not in the IRS (asterisk).

In the hair follicle, GhRL was mainly observed in a specific area, i.e., the suprabulbar region (Figure 2a), while the receptor extended along the entire follicular wall, from the infundibulum to the bulbar region (Figure 2c). GhRL staining was observed in the suprabasal cells of the outer root sheath and in the cells of the inner root sheath (Figure 2b). In contrast, the receptor was present in all layers of the outer root sheath, while the inner root sheath lacked staining (Figure 2d).

**Figure 3:**
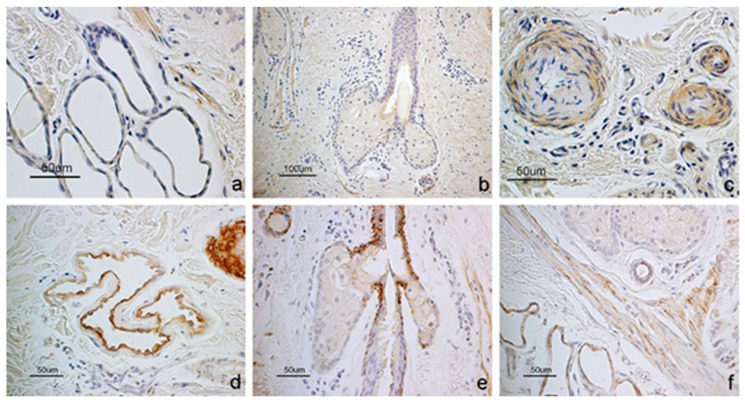
Immunostaining of (**a**–**c**) GhRL and (**d**–**f**) its receptor in the (**a**,**d**) sweat gland, (**b**,**e**) sebaceous gland and (**c**,**f**) smooth muscle cells. (**a**) The sweat and (**b**) the sebaceous glands are negative to GhRL while (**c**) muscle cells of some arterioles appear positive. (**d**) The sweat gland appears positive to GHS-R as well as the arrector pili muscle (**f**) while (**e**) the sebaceous gland is negative.

Sweat glands showed receptor positivity but lacked ligand signal (Figure 3a,d). Sebaceous glands were generally negative for both molecules (Figure 3b,e). However, the receptor was localized in the basal layer of the epithelium lining the short excretory duct connected to the hair follicular epithelium (Figure 3e). In some subjects, the latter positivity extended to the basal cells of the adenomeres. Smooth muscle cells forming the wall of arterioles and the arrector pili muscle were positive to both GhRL and its receptor (Figure 3c,f). The epidermis was negative for both molecules (Figure 2a,c).

Regarding the staining intensity among the sheep groups, the reactions observed in the ghrelin system analysis were consistently strong in all subjects and groups, both in hair follicles and sweat glands. However, the evaluation carried out by score assignment revealed higher positivity for both molecules in the Exp group compared to the MxF and MxD groups. The results are summarized in Table 2.

The Kruskal–Wallis test showed a significant difference in ghrelin staining intensity levels in hair follicle among the three sheep groups (Kruskal–Wallis χ²(2) = 46.84, *p* < 0.001). Post hoc pairwise comparisons using Dunn’s test with Holm correction revealed significant differences between all sheep groups (Table 3).

The ANOVA showed a significant effect of diet on the staining intensity of ghrelin receptors in the hair follicle (F(2, 222) = 30.28, *p* < 0.001).

Post hoc pairwise comparisons using Tukey’s HSD test (Table 4) showed a significant difference between all the groups, with the MxF group associated with the highest reduction in ghrelin receptor intensity compared to the other groups.

The ANOVA showed a significant difference on ghrelin receptor intensity in the sweat glands (F(2,222) = 34,89376, *p* < 0.001).

Post hoc pairwise comparisons using Tukey’s HSD test (Table 5) showed a significant difference between all the groups and, as with the hair follicles, in sweat glands the MxF group resulted in expression of less intense ghrelin receptors staining compared to the Exp and MxD ones.

### 3.2. Real-Time PCR

**Figure 4:**
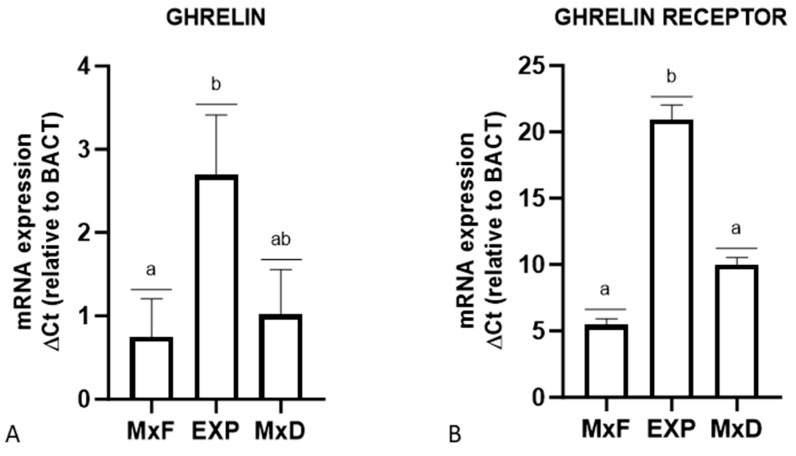
Relative gene expression of (**A**) GhRL and (**B**) GHS-R in the skin of MxF, Exp and MxD sheep groups. The results are expressed as mean mRNA levels 2^−ΔCt^ relative to ACTB and standard deviation values of GhRL and GHS-R. Different letters above the bars indicate significantly different values (*p* < 0.05).

Rt-PCR evidenced the transcript for GhRL and GHS-R in all skin samples analyzed. A significant difference in GhRL expression was observed between Exp vs MxF group, with the Exp group showing a 3.6-fold increase in transcript levels compared to MxF. Ghrelin receptor expression also varied significantly, with the Exp group expressing 3.7-fold more than the MxF group and 2.1-fold more than the MxD group (Figure 4).

## 4. Discussion

The economic sustainability of livestock farms is threatened by increasing summer drought stress linked to global warming [32,33]. The increase of summer aridity negatively impacts the pasture nutritional value. It advances the pasture flowering peak and shortens the period between the maximum flowering and the maximum dryness of grasslands. Therefore, the grazing animals, mainly represented by sheep in the Central Apennine, have increasingly less available and lower quality forage [34,35]. In this scenario, it is of paramount importance to identify effective strategies, such as diet supplementation, aimed at minimizing the above-mentioned negative effects on animal productivity.

In the tested sheep, the GhRL protein was localized in the suprabulbar region of the hair follicles and in the smooth muscle cells of both arterioles and piloerector muscles. The localization of the molecule in the suprabasal cells of the outer root sheath and inner root sheath is consistent with Cicek et al. [36], who described GhRL in human hair follicles. Cicek et al. [36] also observed GhRL in the cell nuclei, cytoplasm and cytoplasmic membrane of the sebaceous gland, but no staining was detected in the sebaceous gland of the sheep. The GhRL receptor showed a wide expression in the sheep hair follicles, being localized throughout the follicular wall from the infundibulum to the bulbar region. Gnanapavan et al. [37] observed that GHS-R1b mRNA is widely expressed in human tissue, with the highest concentration in the skin, while GhRL mRNA has its lowest concentration in the skin compared to the other tissues analyzed [37]. The mRNA pattern described in humans aligns with the immunohistochemical distribution of GhRL and its receptor observed in this study.

The broad localization of the receptor observed in the outer root sheath of the sheep suggests a role of the molecule in hair follicle cell proliferation. Indeed, the outer root sheath contains epithelial stem cells in the bulge region, which proliferate and migrate downwards and upwards as progenitor cells that form a mature hair follicle [38]. This is consistent with what has already been argued by other authors, who suggest that GhRL may be responsible for the growth of hair follicles [39]. Liu et al. [40] showed that GhRL accelerates wound healing in rats in a dose-dependent manner. The ability of GhRL to stimulate proliferation has also been demonstrated in various cells and tissues, including osteoblasts [41], adrenocortical cells [42], cardiomyocytes [43] and several types of cancer cell lines such as prostate [44] hepatoma [45] and colon [46].

In the sheep skin samples, GhRL and its receptor have been identified in the hair follicle, but their immunohistochemical expression only partially overlaps. GhRL was localized in the suprabasal cells of the outer root sheath and the cells of the inner root sheath, while the receptor was present in all cell layers of the outer root sheath. This localization suggests that the molecule may act by both an autocrine and paracrine mechanism in agreement with the mechanism of action of the adipokines in the peripheral tissues [24,47,48,49]. Furthermore, the broad distribution of the receptor along the follicular wall, in contrast to the limited localization of GhRL in the suprabulbar region, suggests that the receptor may also capture circulating GhRL via an endocrine mechanism.

In addition to hair follicle, immunostaining for the receptor, but not for the molecule, was observed in the sheep sweat gland, indicating an endocrine control of its secretory function. Until now, the GHS-R has not been described in normal sweat glands. However, Rozza-de-Menezes et al. [50] observed the GHS-R immunostaining in the human sweat glands of subjects affected by cutaneous neurofibromas. The cells of sweat as well as sebaceous glands have receptors for many molecules, including adipokines [27,51]. It indicates their sensitivity to different physio-pathological conditions, including those related to energy metabolism.

Finally, both GhRL and its receptor were observed in the smooth muscle cells of arterioles and piloerector muscles of the ovine skin. Other authors described a role for GhRL in the modulation of vascular tone and iris sphincter and dilator muscles [52].

In our study, all skin samples taken in the different sheep groups showed the presence of the transcript for the GhRL system. Interestingly, the maximum expression of both GhRL and its receptor was noted in the skin of the Exp group, which consisted of animals that received alimentary supplementation. In contrast, the lowest levels of these molecules were observed in the skin of the MxF groups. These findings were also supported by the statistical evaluation of immunostaining intensities of positive structures to GhRL system molecules. The Exp groups showed a significantly higher intensity for both GhRL and its receptor than the MxD groups, with the lowest intensity showed by MxF groups in both hair follicles and in sebaceous glands. Ghrelin is a molecule that changes in relation to energy metabolism [53], and the functions and effects of GhRL are often related to those of leptin. Aydin et al. [39] showed that obesity induces a decrease in GhRL expression in several rat organs and tissues, including the skin and, specifically, the hair follicle. Accordingly, GhRL expression changes induced by obesity are not limited to the gastrointestinal system organs but involve all the tissues of the organism.

To conclude, both real-time PCR and immunohistochemistry clearly highlight that in the period between maximum pasture flowering and maximum pasture dryness, an increase in GhRL and its receptor occurred in the skin appendages analyzed. It demonstrates that GhRL system functionality is affected by pasture vegetative cycle. In addition, the alimentary supplementation significantly enhanced the expression of the GhRL system molecules, indicating that its functionality is strongly enhanced by alimentary supplementation. During the period tested, the sheep were likely in a phase of follicular growth. In double coat breed sheep, the pattern of fiber growth ranges from a visible molt in the spring to a lesser molt in the autumn that allows the coat to be maintained for the winter [54]. The coat and, accordingly, the hair appearance reflect the status of the hair follicle, which is affected by the body health condition, including metabolism disorders and dietary factors [55,56,57]. As ghrelin may be involved in cellular proliferation and immune protection [16,20,39,42,43], the obtained results allow it to be speculated that the diet supplementation could likely enhance the cyclic activity of the hair follicle as well as the immune protection of this organ through increased expression of ghrelin and its receptor.

## 5. Conclusions

Given the hypotheses on the ghrelin system function on skin, and based on actual findings, it may be speculated that ghrelin is involved in the hair follicle growth in the sheep during the period ranging from the pasture maximum flowering to the maximum dryness. In addition, diet supplementation may positively influence the role of GhRL in promoting the proliferative activity of the hair follicle. The data obtained could be valuable information for breeders who need to be aware of and counteract the negative effects of the increasing summer aridity. This study highlights how diet supplementation can support physiological adaptations in sheep under environmental stress, offering practical applications for improving animal resilience.

## Figures and Tables

**Table 1:** Primers for GhRL, GHS-R and ACTB (used as internal standard) for real-time PCR quantification.

Gene		Primers	bp
GhRL	F	GGAACCTAAGAAGCCGTCAGG	105
	R	ATTTCCAGCTCGTCCTCTGC	
GHS-R	F	CCATCTTCATGCTGGTCGGA	131
	R	GAAGATGCTGGACACCCACA	
ACTB	F	CCTTAGCAACCATGCTGTGA	130
	R	AAGCTGGTGCAGGTAGAGGA	

GhRL: ghrelin; GHS-R; ghrelin receptor; ACTB: beta-actin; bp: base pair.

**Table 2:** Scored staining intensity of positive skin appendages to GhRL and GHS-R in MxF, Exp and MxD groups expressed as mean values ± SD.

Skin Appendage	Tested Molecule	MxF		Exp		MxD	
		Mean	SD	Mean	SD	Mean	SD
Hair follicles	GhRL	2.19	0.63	2.85	0.39	2.43	0.68
	GHS-R	1.71	0.78	2.59	0.53	2.23	0.73
Sweat glands	GhRL	n.d.	n.d.	n.d.	n.d.	n.d.	n.d.
	GHS-R	1.54	0.75	2.49	0.6	2.19	0.78

MxF: Maximum flowering group, Exp: Experimental group, MxD: Maximum dryness group. n.d.: not detected.

**Table 3:** Dunn’s test pairwise comparisons in the ghrelin staining intensity of the hair follicles between the MxF, Exp, and MxD groups.

Comparison	Z	P. Unadjusted	P. Adjusted (Holm)
Exp-MxD	4.13866414	3.49 × 10^−5^	<0.001 ***
Exp-MxF	6.789895389	1.12 × 10^−11^	<0.001 ***
MxD-MxF	2.651231249	0.008019891	0.0080 **

MxF: Maximum flowering group, Exp: Experimental group, MxD: Maximum dryness group. **: *p* < 0.01, ***: *p* < 0.001.

**Table 4:** Tukey’s HSD test pairwise comparisons in the ghrelin receptor staining intensity of the hair follicles between the MxF, Exp, and MxD groups.

Comparison	Difference	Lower	Upper	*p*-Value
MxD-Exp	−0.36	−0.62615	−0.09385	0.0045 **
MxF-Exp	−0.87333	−1.13948	−0.60718	<0.001 ***
MxF-MxD	−0.51333	−0.77948	−0.24718	<0.001 ***

MxF: Maximum flowering group, Exp: Experimental group, MxD: Maximum dryness group. **: *p* < 0.01, ***: *p* < 0.001.

**Table 5:** Tukey’s HSD test pairwise comparisons in the ghrelin receptor staining intensity of the sweat glands between the MxF, Exp, and MxD groups.

Comparison	Difference	Lower	Upper	*p*-Value
MxD-Exp	−0.30667	−0.58158	−0.03175	0.02455 *
MxF-Exp	−0.95333	−1.22825	−0.67842	<0.001 ***
MxF-MxD	−0.64667	−0.92158	−0.37175	<0.001 ***

MxF: Maximum flowering group, Exp: Experimental group, MxD: Maximum dryness group. *: *p* < 0.05, ***: *p* < 0.001.

## Data Availability

The original contributions presented in the study are included in the article; further inquiries can be directed to the corresponding author.
